# Prospective Outcomes of a Biological Resurfacing Arthroplasty with Fascia Lata Autograft (BioJoint) for the Treatment of Osteoarthritis of the Midtarsal Joint Complex

**DOI:** 10.1177/19476035231206740

**Published:** 2023-10-27

**Authors:** Jari Dahmen, Sjoerd A.S. Stufkens, Gino M.M.J. Kerkhoffs

**Affiliations:** 1 Department of Orthopedic Surgery and Sports Medicine, Amsterdam Movement Sciences, Amsterdam UMC, Location AMC, University of Amsterdam, Amsterdam, The Netherlands; 2 Academic Center for Evidence Based Sports Medicine (ACES), Amsterdam UMC, Amsterdam, The Netherlands; 3 Amsterdam Collaboration on Health & Safety in Sports (ACHSS), International Olympic Committee (IOC) Research Center, Amsterdam UMC, Amsterdam, The Netherlands

**Keywords:** osteoarthritis, talonavicular joint, calcaneocuboid joint, biological resurfacing, fascia lata autograft

## Abstract

**Objective:**

To determine the clinical, safety, and radiological outcomes after biological resurfacing arthroplasty for the treatment of osteoarthritis (OA) of one or more joints of the midtarsal joint complex.

**Design:**

All prospectively followed patients with OA to one of or multiple joints of the midtarsal joint complex who were operated with a biological resurfacing arthroplasty with a fascia lata autograft (BioJoint procedure) were included. A total of 7 patients were included (5 males, 2 females), with a median age of 52 (interquartile range [IQR] 44-55) years. The primary outcome was the Numeric Rating Scale (NRS) for pain during walking 2 years postoperatively. Range of motion (ROM), revision rates, and complications were assessed. A postoperative MRI scan was performed to assess progression of OA, graft position and ingrowth, and the degree of bone marrow edema.

**Results:**

There was a 100% follow-up of the patients (median follow-up: 33 [IQR 26-33] months). The NRS during walking improved from 6 preoperatively to 2 at 2 years postoperatively (*P* < 0.05). There were no reoperations nor severe complications. The limitations in the ROM remained limited in the majority of the cases. MRI at 2 years of follow-up showed no progression of OA, reduced bone marrow edema, and no loosening of the grafts.

**Conclusion:**

Biological resurfacing arthroplasty with a fascia lata autograft (BioJoint procedure) for OA to one or more joints in the midtarsal joint complex showed clinically relevant pain reduction during walking, improvement in clinical and radiological outcomes, and proved to be safe and durable.

## Introduction

Osteoarthritis (OA) to one or more of the joints of the midtarsal joint complex is a common posttraumatic problem.^1,2^ Patients mainly complain of pain during bearing weight and stiffness with a potentially restricted motion. The non-operative treatment options can consist of stabilization of the affected joint by wearing a brace or modified footwear, activity modification, weight loss, steroid or hyaluronic injections, pain medication, and physiotherapy.^2,3^ When conservative treatment fails, (more invasive) surgical measures can be discussed with the patient.^4^

When considering surgical treatments for OA to the joints of the midtarsal joint complex, arthroscopic or open debridement may be helpful in the early stages, during which damaged cartilage, loose cartilage, and bony fragments or osteophytes can be removed.^5-7^ The current definite surgical treatment is a fusion of the affected joint.^8-11^ A fusion is a joint-sacrificing surgery that leads to loss of mobility (range of motion, ROM) of the joint in question with the possible consequence of an impaired gait pattern as well as the overload of adjacent joints. Moreover, good options for metal arthroplasty for the joints located within the midtarsal joint complex are currently absent.^3^

As such, the development of alternative surgical treatment options preserving the motion of the affected joint with a combinatory effect of adequate pain reduction is wished for. One of these joint-preserving treatment options includes a biological resurfacing arthroplasty providing mechanical support and coverage of the affected articular surfaces without compromising biomechanical and biochemical properties with a preservation of the joint’s ROM. From a clinical perspective, these newly developed surgical treatment options are therefore suited for the surgical treatment of OA to one or more of the joints of the midtarsal joint complex and have promising clinical results when considering other joints.^12,13^ One of the examples of biological resurfacing arthroplasties is the usage of an autograft from the ipsilateral fascia lata.^14^

There are currently no studies on the application of this specific procedure for OA to one or multiple joints within the midtarsal joint complex. It is therefore the purpose of the present study to investigate the clinical efficacy of the interposition fascia lata autograft (also referred to as the BioJoint procedure) for the treatment of OA located in the talonavicular, calcaneocuboid (CC), and the other small joints of the midtarsal joint complex. It is hypothesized that the postoperative clinical outcomes concerning pain and implant autograft survival will be good.

## Methods

This is a prospective, single-center, nonrandomized, clinical cohort study with a 2-year follow-up. The study was approved by the local Medical Ethics Committee Approval at the Amsterdam UMC, location AMC (reference number: MEC 08/326), and the study was performed in accordance with the Declaration of Helsinki. Our center is a tertiary referral academic hospital accredited as nationally and internationally recognized center for the diagnosis and treatment of cartilage and osteochondral lesions of the foot and ankle.

### Patient Selection

All patients with OA to one of or multiple joints of the midtarsal joint complex (i.e. the CC joint, the talonavicular joint, and/or the inter-cuneiform joint(s) medial) who were operated with the biological resurfacing arthroplasty (BioJoint procedure) from December 2018 onward and had a prospective minimal follow-up of 24 months were included in the present study. The recruitment of patients was performed as usual with all patients on the outpatient clinic at the Department of Orthopedic Surgery at the Amsterdam UMC—location AMC taking into account a thorough shared decision-making process. The inclusion and exclusion criteria are to be appreciated in **Table 1**. A total of 7 patients with an insertion of 8 BioJoints were eligible for inclusion—one patient had a BioJoint insertion in both the talonavicular joint and the joint between the intermediate cuneiform and medial cuneiform. Patient demographics are summarized in **Tables 1** and **2**. Five males and 2 females with a median age of 52 (interquartile range [IQR] 44-55) years were included.

**Table 1.** Inclusion and Exclusion Criteria.

Inclusion Criteria	Exclusion Criteria
Radiograph and/or CT-confirmed osteoarthritis to one of or multiple joints of the midtarsal joint complex (i.e. the calcaneocuboid joint, the talonavicular joint, and/or the inter-cuneiform joint(s) medial)	Diabetes mellitus/rheumatoid osteoarthritis
Willing to receive surgical implantation of the fascia lata autograft (BioJoint procedure) and age ranging from 18 to 80 years	Advanced osteoporosis
The subject and treating physician agree that the subject will return for all required post-procedure follow-up visits	Blood supply limitations and previous infections, which may retard healing
Complaints for at least 6 months	Active infection or blood supply limitations
Failed previous conservative or surgical treatment, including prior joint fusion	Conditions that tend to limit the patient’s ability or willingness to restrict activities or follow directions during the healing period, including severe neuro-arthropathy
Minimum follow-up of at least 24 months	Pathological conditions, such as insufficient quantity or quality of bone (severe osteopenia), which may compromise implantation of the fascia lata autograft

CT = computed tomography.

**Table 2.** Patient Demographic Factors.

Joint	Patient	Age at Surgery (Years)	BMI (kg/m^2^)	Number of Prior Surgeries to the Foot/Ankle	Type of Prior Surgeries/Surgery	Gender	Alignment	Follow-up	Concomitant Surgery	Indication
Calcaneocuboid	1	51	30	1	Open nettoyage of osteochondral defect to the calcaneocuboid bone and removal of an avulsion fragment Processus anterior calcanei	F	Neutral	25 months	None	Posttraumatic osteoarthritis to the calcaneocuboid joint
Calcaneocuboid	2	18	23	1	External referral hospital: open excochleation of an osteoblastoma to the cuboid bone	M	Neutral	41 months	Yes—excochleation of a recurrent osteoblastoma of the cuboid bone	Osteoarthritis due to destruction of the calcaneocuboid joint because of recurrent osteoblastoma to the cuboid bone
Calcaneocuboid	3	63	31	3	Surgery 1 (external referral hospital): elongation of the calcaneus with a concomitant gastric slide Surgery 2 (external referral hospital): release of peroneal tendons and removal of osteosynthesis material after calcaneus elongation Surgery 3: removal of osteophyte to the hallux	M	Neutral	33 months	Yes—posterior ankle arthroscopy for removal of subtalar osseous impingement	Isolated osteoarthritis to the calcaneocuboid joint
Calcaneocuboid	4	53	34	1	Placement of an external fixator due to a post-traumatic communicated cuboid fracture with delayed union (due to persistent swelling, no definitive osteosynthesis performed at external referral hospital)	M	Neutral	26 months	None	Posttraumatic osteoarthritis to the calcaneocuboid joint
Talonavicular and joint between medial cuneiform and intermediate cuneiform	5	BioJoint surgery 1 (unfusion of the naviculocuneiform and talonavicular joint and insertion of BioJoint into the talonavicular joint): 43 BioJoint surgery 2 (between medial cuneiform and intermediate cuneiform): 44	31	3	Surgery 1: bilateral clubfoot correction Surgery 2: (referral hospital) planned talonavicular joint arthrodesis resulted in naviculocuneiform joint arthrodesis (Lisfranc fusion) Surgery 3: (referral hospital) arthrodesis of the talonavicular joint	M	Mild pes cavovarus	33 months	None	See also “type of prior surgeries” Indication BioJoint 1: (failed/incorrect) fusion of naviculocuneiform joint and the talonavicular joint Indication BioJoint 2: osteoarthritis to the joint between the medial cuneiform and intermediate cuneiform
Talonavicular	6	55	29	0	NA	M	Mild pes cavovarus	30 months	None	Isolated non–post-traumatic osteoarthritis talonavicular joint
Talonavicular	7	55	29	0	NA	F	Mild pes cavovarus	29 months	None	Isolated non–post-traumatic osteoarthritis talonavicular joint

BMI = body mass index; NA = not available; F = female; M = male.

### Preoperative Planning

As part of our clinical workup for patients with OA to joints of the foot and/or ankle, all patients received a baseline dual-energy computed tomography scan to assure the diagnosis and to determine the extent and exact localization of the OA. After assuring the diagnosis, the patients were provided with a comprehensive and thorough individualized shared decision-making process during which the different treatment options are discussed in an interactive and dynamic manner. One of the treatment options that is included in the treatment algorithm is the implantation of the fascia lata autograft (BioJoint procedure). As part of our treatment algorithm, all patients receive all possible information about the different treatment options, including the surgical procedures, rehabilitation, and advantages and disadvantages of each treatment option. Patients were included in the present trial whenever the patients chose together with the treating physician to opt for the BioJoint procedure.

### Surgical Technique and Postoperative Management

All procedures were performed by two experienced fellowship-trained academic foot and ankle orthopedic surgeons (G.M.M.J.K. and S.A.S.S.). All patients undergoing the interposition arthroplasty with fascia lata autograft (BioJoint procedure) received preoperative antibiotics (cefazoline 2 g intravenous) according to the regular protocol. The procedure was carried out under general or spinal anesthesia. The patient was placed in the supine position with a tourniquet applied near the groin around the thigh. The sole of the foot was aligned with the end of the table, and a soft bump was placed under the ipsilateral hip to internally rotate the leg. The whole leg was disinfected and sterile draping of the surgical field was performed to allow for graft harvesting of the ipsilateral leg.

The approach to the talonavicular, CC, and inter-cuneiform joints was dependent on the joint. For the talonavicular joint, an anteromedial approach was used through an L-shaped incision of the capsule. For the CC joint, a slightly curved lateral approach was used over the CC joint, and for the inter-cuneiform joint(s), a centrolateral approach was used over the joint just laterally from the anterior tibial tendon (the exact osseous location of the medial cuneiform and intermediate cuneiform was assessed through fluoroscopy). In short, the exposure was continued after which present osteophytes, fibrous tissue, and diseased cartilage not being part of the former natural joint were removed in order to create space for the insertion of the graft. Then, a debridement of the diseased joint surface, and the subchondral bone took place in order prepare to allow ingrowth of the graft. A Hintermann spreader was placed to open and distend the joint in a gradual fashion taking into account the tension on the soft tissue surrounding the joint. Once the exposure was finalized, a flexible ruler was used to measure what the intended size of the interpositional autograft was by assessing the surface of the particular joint. Actual sizing of the autograft should be approximately 2-3 mm larger in all directions to allow for proper surfacing.

**Figure 1.**
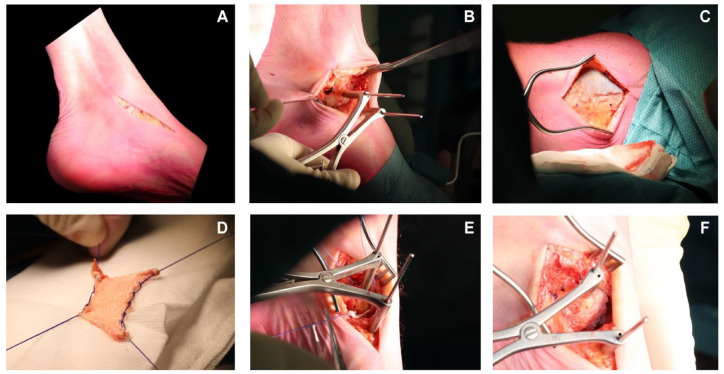
Intra-operative images of talonavicular BioJoint resurfacing. (**A**) Anteromedial incision. (**B**) Distending the talonavicular joint with the Hintermann spreader. (**C**) Attention toward the fascia lata with the blue dots indicating the edges of the graft. (**D**) After harvesting the fascia lata biological resurfacing autograft. (**E**) Positioning the autograft into the joint prior to the final knotting phase. (**F**) Final result of the joint after the inserted autograft.

**Figure 2.**
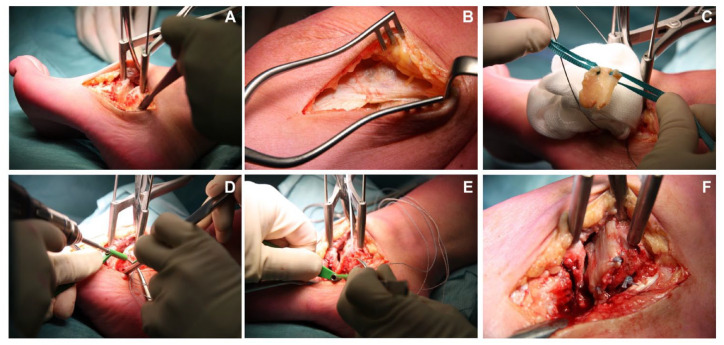
Intra-operative images of a calcaneocuboid BioJoint resurfacing. (**A**) After approaching the calcaneocuboid joint with a distension of the joint with the Hintermann spreader. (**B**) Attention toward the ipsilateral fascia lata with image (**C**) being the result after harvesting the autograft and having started the process of braiding in the first two edges of the graft. (**D** and **E**) Drilling 2 tunnels (1 superior and 1 inferior) into the calcaneus. (**F)** Final result of the joint after the inserted autograft.

The graft was then harvested from the ipsilateral leg. An incision was made just superiorly to the lateral condyle of the femur after which the vision was exposed onto the distal fascia lata. This fascia lata was excised in a diamond-shaped manner as sized to the receiving joint. The fascia lata was not released distally and dissection stayed superficial to the joint capsule, prohibiting arthrotomy. After harvesting of this graft, the layers were be closed, after which a pressure bandage was applied. The graft was harvested according to the measured dimensions so that the desired size for the resurfacing of the joint was reached. Two tunnels were then drilled through the talar neck (in case of the talonavicular BioJoint), through the calcaneal side (in case of the calcaneocuboid BioJoint), or through the proximal and distal side of the medial cuneiform (in case of the BioJoint between the medial and intermediate cuneiform) with a 2.5-mm drill after which the graft was pulled into all 4 corners of the involved joint. The graft was inserted, and subsequently retrieved with a suture anchor retriever (e.g. using the Arthrex suture anchor [Naples, FL] suture anchor, or in cases with limited working space a PDS loop); adequate tension was put on the graft, and the sutures were then knotted in a transosseous fashion on the graft itself. The final placement of the graft was checked in all the 4 corners, after which the ROM was checked to ensure the graft was accurately fixated and did not impinge nor translate. Afterward the incision was closed in layers. A compressive dressing was applied, as well as a lower-leg splint or a Walker. Intra-operative images for the talonavicular procedure and the CC procedure are included in **Figures 1** and **2**.

### Postoperative Treatment

After 2 weeks of not bearing weight in the lower-leg splint, the lower-leg splint is replaced for a removable splint. The patient is then allowed and motivated to start ROM exercise. Active ROM and passive ROM are supervised by an experienced foot and ankle physiotherapist. After 6 weeks the patient is allowed to start bearing weight.

### Clinical Evaluation

Standard preoperative patient demographics were extracted. Preoperative and postoperative (2 years of follow-up postoperatively) assessment consisted of an electronic questionnaire consisting of the following patient-reported outcome measures (PROMs): the Numeric Rating Scale^15^ (NRS) of pain during walking (primary outcome), at rest, during running, and during stairclimbing; the Foot and Ankle Outcome Score^16^ including its subscales; and the Short-Form 36 Mental Component Scale and Physical Component Scale.^17^ To minimize the risk of bias, the treating orthopedic surgeons were not involved in any outcome assessment. For all questionnaires, a version validated in the native language was used.^16-19^ Subjective satisfaction with the outcome of the procedure was additionally asked and noted in a binary manner (yes/no) at 2 years of follow-up. Other clinical outcomes consisted of a prospective assessment of occurrence of additional surgical procedures, complications, and presence of donor-site morbidity—patients were actively requested to report on the presence of donor-site morbidity at 2 years follow-up being defined as pain, disability, or hindrance in or around the operated area affecting activities in daily life. Moreover, patients were asked whether there were (painful) complaints of the donor site in the first 3 months. Another secondary parameter is the measurement of postoperative ROM with a handheld goniometer and will be registered in absolute difference in degrees of ROM as compared to the contralateral joint.

### Radiological Evaluation

Concerning the postoperative radiological assessment of the patients, 3 different outcomes were assessed through a binary outcome assessment manner at the postoperative MRI scan by an independent experienced musculoskeletal radiologist: (1) reduction of degree of bone marrow edema as compared to the preoperative MRI (yes/no)—in case there was no preoperative MRI available, a comparison to the first postoperative MRI was performed, (2) loosening of the inserted autograft (yes/no), and (3) signs of progression of OA (yes/no) as assessed by the presence of potential enlargement of cysts, progression of subchondral sclerosis, joint space narrowing and as compared to any preoperative radiological entities.

### Statistical Analyses

The primary outcome was defined as the difference between the preoperative and postoperative (2 years) NRS^10^ during walking. All other outcomes were regarded as secondary outcome measures. All statistical analyses were performed with SPSS version 28.0 (SPSS Inc., Chicago, IL). Dichotomous and categorical outcome variables were reported as absolute numbers and percentages. Continuous outcome variables were reported as means and standard deviations in case of normal distribution of the data and as median and IQR in case of non-normally distributed data. The ROM of the talonavicular joint was measured in degrees of dorsiflexion as well as plantarflexion as a combinatory value from the talocrural and talonavicular joint, while the ROM from the CC joint was measured in degrees of inversion and eversion from the subtalar joint. Whenever there was a deficit in the number of degrees, this was noted with a minus sign. Preoperative and postoperative data were compared with a paired *t* test in case of normalized data and with a Wilcoxon signed-rank test in case of non-normally distributed data. When the pre-operative value was non-normally disributed and the post-operative value was (or vice versa), means were used as statistical representation of the data. The level of significance was set at *P* < 0.05.

## Results

### Patient Selection and Demographics

No patients were lost to follow-up (100% follow-up). The median follow-up was 33 (IQR 26-33) months. Patient-specific details and outcomes are summarized in **Tables 2** and **3**.

**Table 3.** Outcomes.

Joint	Patient	Satisfaction	Complaints Donor Site First 3 Postoperative Months	Complaints Donor Site 2 Year Postoperatively Affecting Daily Life Activities	Range of Motion (Degrees of Difference Compared to Contralateral Side)		MRI Outcome		
							Reduction of Degree of Bone Marrow Edema as Compared to First Postoperative or Preoperative MRI	Loosening of the Graft at 2 Years of Follow-up	Progression of OA
Calcaneocuboid	1	Yes	No	No	Inversion: −5° difference	Eversion: −7° difference	Yes (no preoperative MRI)	No	No
Calcaneocuboid	2	Yes	Yes	No	Inversion: −15° difference	Eversion: −3° difference	Yes	No	No
Calcaneocuboid	3	Yes	Yes	No	Inversion: −25° difference	Eversion: −10° difference	Yes (no preoperative MRI)	No	No
Calcaneocuboid	4	Yes	Yes	No	Inversion: NA	Eversion: NA	Yes	No	No
Talonavicular & joint between medial cuneiform & intermediate cuneiform	5	Yes	Yes	No	Dorsiflexion: −0° difference	Plantarflexion: −0° difference	No	No	No
Talonavicular	6	Yes	No	No	Dorsiflexion: −2° difference	Plantarflexion: −0° difference	Yes	No	No
Talonavicular	7	Yes	No	No	Dorsiflexion: −2° difference	Plantarflexion: −0° difference	Yes	No	No

NA = not available; OA = osteoarthritis.

### Patient-Reported Outcome Measures

The primary outcome, the NRS of pain during walking, decreased from a mean of 5.7 (SD: 2.5) preoperatively to a mean of 2.0 (SD: 1.9) postoperatively (*P* < 0.05). The secondary outcomes including the (other) NRS scores and PROMs are (also) presented in **Table 4**. Hundred percent of the patients were satisfied with the outcome **(Table 3)**.

**Table 4.** Patient-Reported Outcome Measures.

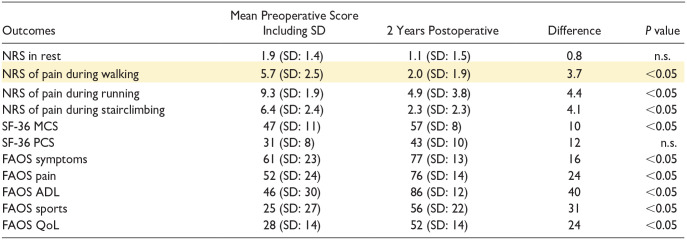

FAOS = Foot and Ankle Outcome Score; SD = standard deviation; MCS = Mental Component Scale; NRS = Numeric Rating Scale; n.s. = not significant (yellow indicates primary outcome); PCS = Physical Component Scale; SF = Short-Form; QoL = quality of life; ADL = activities in daily living.

### Radiological and ROM Outcomes

The radiological and ROM outcomes are presented in **Table 3**.

### Additional Surgical Procedures, Donor-Site Morbidity, and Complications

From the baseline surgery to the 2 years of follow-up, no patients (0%) required a re-operation to the involved joint. As previously noted, one patient underwent an additional BioJoint insertion to the talonavicular after the prior BioJoint insertion between the medial cuneiform and the intermediate cuneiform. There were no severe complications (0%). Two patients (29%) developed minor complications: One patient developed fasciitis plantaris postoperatively and one patient developed bursitis subtrochanterica postoperatively.

Fifty-seven percent of the patients complained of donor-site morbidity in the first 3 months after the surgery. No patient (0%) reported donor-site morbidity complaints at 2-year follow-up that was classified as pain, disability, or hindrance in or around the operated area affecting activities in daily life **(Table 3)**. There was one (14%) patient who complained of donor-site “cramping” at 2 years of follow-up; however, this was not classified by the patient as complaints that affected daily life activities.

## Discussion

In our prospective study, the biological resurfacing arthroplasty procedure, also referred to as the BioJoint procedure, proved to be a clinically effective and safe technique for the treatment of end-stage OA to one or more of the joints of the midtarsal joint complex. A median improvement of 4 points on the NRS of pain during walking is considered highly effective since a mean improvement of −2.0 on the NRS scale of 0-10 is significantly associated with a “much better improvement” in pain and, as such, reaches beyond the minimal clinically important difference (MCID).^20^

First, our study highlights the positive impact of the novel BioJoint procedure on the patients’ complaints. Although not fully new (as the procedure could be considered a derivative of a suspension arthroplasty or Sardella plasty which was described a few decades ago for carpometacarpal OA^21^), the improvements in the clinical outcomes as assessed with our outcomes, and especially the PROMs, further support the efficacy of the present treatment option. The findings of the pain reduction are consistent with other surgical treatment options for OA to the joints of the midtarsal joint complex.^8-11^ However, when comparing our results to the current definite surgical treatment (i.e. arthrodesis) of OA in the joints within the midtarsal joint complex, the disadvantages of an arthrodesis should be taken into account as well although we know that a subtalar and a talonavicular joint fusion leads to excellent union rates as well as adequate reductions in pain.^22^ It should be mentioned in this context that an arthrodesis is a joint-sacrificing, irreversible surgical treatment option in which the mobility (ROM) of the joint in question is lost which can lead to an impaired gait pattern as well as overload of adjacent joints in the foot and/or ankle with subsequent degeneration as well.^23^ With regard to this, it can be discussed that patients with a CC joint fusion may have persisting complaints due to an impaired walking pattern and roll-off of the foot—this can be partially due to the fact that the complete loss of the subtalar joint motion will make it difficult for patients to adapt to uneven surfaces and limit activities involving inversion and eversion. This was substantiated by a study of Usuelli *et al.*^24^ finding that the walking speed and stride of patients who underwent subtalar joint fusion in combination with ankle arthroplasty were significantly lower compared to a healthy control group in patients who underwent subtalar joint fusion in combination with ankle arthroplasty noting that the fusion of the subtalar joint appeared to be compensated by larger frontal-plane motion at the ankle joint. In a similair study by Wu et al.25 with an experimental set-up including patients with either a triple arthrodesis or a subtalar fusion, the authors found that the operated limbs showed a different moveement pattern with decreased hip flexion moments, decreased ankle plantarflexion moments as well as lower power generation during the push-off phase. An older study by Fogel *et al.*^26^ dating back from 1982 revealed similar outcomes when performing an objective gait analysis on patients having undergone a talonavicular (isolated) fusion: It was shown that there was a decrease in plantarflexion at the lift-off period of the gait cycle as well as a decreased subtalar joint motion when the patients were performing the pronation movements. As such, these publications may explain why patients may benefit from a BioJoint insertion with more magnitude with regard to the clinical and walking pattern outcomes in comparison to a fusion.^22,27^

Besides the description and discussion of the clinical outcomes, it is important to describe the presence of potential donor-site morbidity after harvesting of the graft on the ipsilateral side of the upper leg. In our prospective study, the patients were specifically asked to report donor-site morbidity of the harvesting site; none (0%) reported functional impairments due to donor-site morbidity. However, a specific donor-site morbidity assessment was also performed in the first 3 months after the surgery, and it was noted that 57% of the patients reported complaints around the donor site in this time period. It is worth mentioning that one patient (14%) complained of donor-site “cramping” at the 2-year follow-up, but it did not substantially impact daily life activities. To prevent these donor-side problems, an alternative graft would be a decellularized dermal matrix such as the ArthroFLEX^®^ (Arthrex Inc., Naples, FL). The ArthroFLEX^®^ has been successfully used for various other indications, such as for acromioclavicular joint resurfacing, interposition arthroplasty for the ankle joint, as well as in patients with degeneration of the wrist due to OA.^28-30^ Concerning the complication assessment, there were no major complications though minor complications were observed in two patients (29%), including fasciitis plantaris and bursitis subtrochanterica. These complications were manageable and did not require additional surgical interventions. Moreover, no patients required additional surgeries to the graft nor did any (0%) of the patients require a revision to an arthrodesis during the first 2 years of this prospective follow-up. As such, it can be concluded that the procedure does not cause any harm concerning donor-site morbidity and can be regarded a safe and viable procedure—especially in relation to the publication occurrence after surgical treatment for (end-stage) OA to joints in the midtarsal joint complex which may entail nerve injuries, infections, non-unions, or delayed unions (in case of fusion of the joint).

Although the outcomes of our study can be considered promising, our study is limited to a single arm, and joints solely included the ones related and included in the midtarsal joint complex. However, we have recently shifted the indication setting to OA to the ankle joint as well as to the metatarsophalangeal joint(s). Another limitation is the relatively low small sample size. Consequently, further and future studies with larger cohorts and longer follow-up times need to be undertaken to further validate the results. Strengths of the present article are the prospective nature, the 100% follow-up rate, and the usage of a diverse set of clinical outcome scores with an in-depth and per-patient qualitative approach to the interpretation of the demographic and outcome factors.

From a clinical relevance point of view, we can state that when managing patients with OA in the midtarsal joint complex, this biological resurfacing arthroplasty (BioJoint procedure) may represent a viable joint-sparing alternative to invasive joint-sacrificing surgeries—such as an arthrodesis surgery. By preserving the joint, this technique may potentially extend joint longevity and delay or even obviate the need for more aggressive surgical interventions. Patients who might have otherwise faced the prospect of extensive joint sacrifice can now benefit from a treatment option that not only provides pain relief and functional improvement but also maintains joint integrity.

As with any novel procedure, careful patient selection and appropriate preoperative evaluation are critical to ensuring optimal outcomes. By considering the benefits observed in this study, clinicians can potentially incorporate the biological resurfacing arthroplasty into their treatment arsenal when applying a shared decision-making algorithm providing patients with a tailored approach that addresses their specific needs and offers the potential for enhanced long-term joint function and overall quality of life. Further research and larger scale studies will be valuable in confirming the long-term efficacy and safety of this technique, solidifying its place as a valuable addition to the array of treatments available for OA in the midtarsal joint complex.

### Conclusion

The implantation of a biological resurfacing arthroplasty for OA (the BioJoint procedure) to one or more of the joints included in the midtarsal joint complex showed a clinically relevant pain reduction during walking, improvement in clinical and radiological outcomes as assessed with PROMs and follow-up MRIs, and no complications nor re-operations. This treatment option can evolve as a joint-sparing alternative to invasive joint-sacrificing surgeries.

## Supplemental Material

sj-docx-1-car-10.1177_19476035231206740 – Supplemental material for Prospective Outcomes of a Biological Resurfacing Arthroplasty with Fascia Lata Autograft (BioJoint) for the Treatment of Osteoarthritis of the Midtarsal Joint Complex Supplemental material, sj-docx-1-car-10.1177_19476035231206740 for Prospective Outcomes of a Biological Resurfacing Arthroplasty with Fascia Lata Autograft (BioJoint) for the Treatment of Osteoarthritis of the Midtarsal Joint Complex by Jari Dahmen, Sjoerd A.S. Stufkens and Gino M.M.J. Kerkhoffs in CARTILAGE
