# Supplementary material for: Prospective Outcomes of a Biological Resurfacing Arthroplasty with Fascia Lata Autograft (BioJoint) for the Treatment of Osteoarthritis of the Midtarsal Joint Complex
Source: Cartilage. 2023 Oct 27;15(1):37–46. doi: 10.1177/19476035231206740 (PMC10985392; doi:10.1177/19476035231206740)
Supplement: sj-docx-1-car-10.1177_19476035231206740 – Supplemental material for Prospective Outcomes of a Biological Resurfacing Arthroplasty with Fascia Lata Autograft (BioJoint) for the Treatment of Osteoarthritis of the Midtarsal Joint Complex [file sj-docx-1-car-10.1177_19476035231206740.docx]

**RESPONSES TO THE PEER-REVIEWS**

**Comment 1:**

The title ¨Prospective Outcomes of a Novel Biological Resurfacing Arthroplasty (BioJoint) for the Treatment of Osteo-Arthritis of the Mid-Tarsal Joint Complex¨might be slightly changed to: Prospective Outcomes of resurfacing arthroplasty with fascia lata graft (BioJoint) for the Treatment of Osteo-Arthritis of the Mid-Tarsal Joint Complex.

**Response to comment 1:**

Done.

**Comment 2:**

Add information of the use of fascia lata graft in abstract methods: In Methods only BioJoint is mentioned but in the text it should be: Fascia lata graft resurfacing (BioJoint).

**Response to comment 2:**

Performed in the abstract and main text.

**Comment 3:**

In Methods, age and gender of study patients should be mentioned. In results: Mean follow up time should be included and similar in the abstract.

**Response to comment 3:**

Performed in the abstract and main text.

**Comment 4:**

When mentioning products like Arthroflex info about producer and country is needed

**Response to comment 4:**

Thank you for the comment. This has been incorporate.

**Comment 5:**

When mentioning Biojoint, I suggest that the authors name it instead¨ Biojoint procedure¨. It is otherwise easy to misinterpret the title that it is product implant rather than autologous or allogeneic biological tissue

**Response to comment 5:**

Thank you for the comment. This has been changed all over the manuscript.
